# High-Throughput Sequencing and *De Novo* Assembly of the *Isatis indigotica* Transcriptome

**DOI:** 10.1371/journal.pone.0102963

**Published:** 2014-09-26

**Authors:** Xiaoqing Tang, Yunhua Xiao, Tingting Lv, Fangquan Wang, QianHao Zhu, Tianqing Zheng, Jie Yang

**Affiliations:** 1 College of Horticulture, Nanjing Agricultural University, Nanjing, The People’s Republic of China; 2 Institute of Food Crops, Jiangsu Academy of Agricultural Sciences, Nanjing, The People’s Republic of China; 3 CSIRO Plant Industry, Canberra, Australia; 4 Institute of Crop Sciences, Chinese Academy of Agricultural Sciences, Beijing, The People’s Republic of China; Case Western Reserve University, United States of America

## Abstract

**Background:**

*Isatis indigotica*, the source of the traditional Chinese medicine Radix isatidis (Ban-Lan-Gen), is an extremely important economical crop in China. To facilitate biological, biochemical and molecular research on the medicinal chemicals in *I. indigotica*, here we report the first *I. indigotica* transcriptome generated by RNA sequencing (RNA-seq).

**Results:**

RNA-seq library was created using RNA extracted from a mixed sample including leaf and root. A total of 33,238 unigenes were assembled from more than 28 million of high quality short reads. The quality of the assembly was experimentally examined by cDNA sequencing of seven randomly selected unigenes. Based on blast search 28,184 unigenes had a hit in at least one of the protein and nucleotide databases used in this study, and 8 unigenes were found to be associated with biosynthesis of indole and its derivatives. According to Gene Ontology classification, 22,365 unigenes were categorized into 48 functional groups. Furthermore, Clusters of Orthologous Group and Swiss-Port annotation were assigned for 7,707 and 18,679 unigenes, respectively. Analysis of repeat motifs identified 6,400 simple sequence repeat markers in 4,509 unigenes.

**Conclusion:**

Our data provide a comprehensive sequence resource for molecular study of *I. indigotica*. Our results will facilitate studies on the functions of genes involved in the indole alkaloid biosynthesis pathway and on metabolism of nitrogen and indole alkaloids in *I. indigotica* and its related species.

## Introduction

*Isatis indigotica* Fort. (Chinese woad) is a biennial herbaceous medicinal plant species distributed widely in China. Isatidis Folium and Isatidis Radix, two common Chinese medicines, are leaves and roots of *I. indigotica*, respectively. Their common names are “Da-Qing-Ye”and “Ban-Lan-Gen”, respectively, in the Chinese Pharmacopeia [1]. Previous studies have shown that Da-Qing-Ye and Ban-Lan-Gen have a wide range of pharmacological bioactivities, including antivirus [2], [3], [4], anti-bacterial [5], anti-endotoxic [6], antitumor [7], anti-inflammatory [8], [9] and immune regulatory effects [10]. Chemical research indicated that Da-Qing-Ye contains a substantial amount of alkaloids [4], [11], [12], [13], organic acids [14], nucleosides [15], ignanoids [16], quinoline and quinazolone [17].

The clinical and pharmacological properties of *I. indigotica* attracted investigations on the cellular biochemical and biological aspects of latex biogenesis in this economically important medicine plant. In order to gain insights into biosynthesis of alkaloids, the gene encoding alpha-tryptophan synthase has been cloned and analyzed in *I. tinctoria*, a species closely related to *I. Indigotica*, although they differ in several morphological traits [18]. In addition, *I. tinctora* was widely cultivated in certain areas of Europe, such as Italy, from the 12^th^ to the 17^th^ century [19], and was used as dye-plant to extract indigo[20], but *I. indigotica*, first described by Fortune (1846) [21], has not been widely used as a dye-plant although it contains same indigo precursors as its European counterpart *I. tinctoria*.

So far only about 100 nucleotide sequences and 50 expressed sequence tags (ESTs) are available for *I. tinctoria* in the GenBank (the National Center for Biotechnology Information).The number of available nucleotide sequences of *I. indigotica* is even less than that of *I. tinctora*. Moreover, little information is current available for functional genes in *I. tinctoria* and *I. indigotica*. The genetic transformation efficiency of *I. indigotica* has been significantly improved recently, which is expected to promote the breeding process of *I. indigotica* using a genetically modified approach. However, lack of genomic sequences, particularly sequences of functional genes, makes it now impossible to improve the performance of *I. indigotica* by gene transformation. It is thus necessary to discover and characterize transcriptome of *I. indigotica*.

Transcriptome sequencing or RNA sequencing (RNA-seq) is one of the recently developed high-throughput sequencing methods that are able to produce millions of short cDNA reads in a parallel manner. RNA-seq can be used to determine sequences and abundance of transcripts, even at the single-cell level [22]. RNA-seq has been widely used in characterization of transcriptomes in model plant species, such as rice and *Arabidopsis*. It has also been successfully used in identification of alternatively spliced transcripts and long non-coding RNAs responsive to stresses in *Arabidopsis* [23], [24]. A holistic view of a transcriptome can be offered by RNA-seq, including novel transcriptionally active regions and the precise location of transcription boundaries [25]. RNA-seq is especially useful for analysis of transcriptomes of non-model species [26]–[28] as no prior knowledge of transcript sequence is need.

Simple sequence repeat (SSR) is one of the most commonly used molecular markers in genetic mapping and gene fingerprint in plants; however, no SSRs are current available in *I. indigotica*, which confines studies of quantitative traits in this important medicinal plant. Transcriptome is an important sequence resource for identification of SSRs and has been frequently used in SSR identification in plants [29].

The major goal of this study was to generate the transcriptome of *I. indigotica* using RNA-seq and to annotate the transcriptome using publicly available databases and tools. Our main focus was to determine the genes involved in biosynthesis of indole and its derivatives, which are also possibly related to nitrogen metabolism. To this end, messenger RNAs (mRNA) isolated from leaves and roots of the vigorously vegetative growth stages were used in creation of RNA-seq library. Unigenes were *de novo* assembled from the sequenced short reads. The unigenes were then annotated by BLASTX search, Gene Ontology (GO) classification and pathway analysis using the Kyoto Encyclopedia of Genes and Genomes (KEGG). Our work demonstrated the suitability of RNA-seq in *de novo* assembly and annotation of *I. indigotica* genes. Our results also provide a foundation for further functional characterization of *I. indigotica* genes.

## Results

### *De novo* assembly of transcriptome and generation of unigenes

To obtain a comprehensive set of *I. indigotica* transcripts, RNA-seq was performed by using mRNA isolated from leaves and roots of *I. indigotica*. Leaves were collected from 8∼16-leaf stage plants and roots were collected from 14∼16-leaf stage plants, respectively.

We generated 28,283,587 original reads with a total 5.71 Gbp nucleotides using an Illumina Hiseq 2000 sequencing machine. We used NGSQCToolkit (v2.3) [30] and a set of stringent criteria to remove the low quality paired-end reads or reads containing adaptors. After this quality control (QC) step, 23,658,849 clean and high quality reads (101 bp in length) with a total of 4.78 Gbp nucleotides were retained for further analysis. These reads were used to assemble contigs and then transcripts using the Trinity program with the default parameter settings [30]. In total, 1,189,038 contigs were generated with a *k*-mer of 25, which was pre-defined in the program to avoid mis-assembly caused by too short *k*-mer [31], [32] but to retain a decent number of reads in the assembly. From these contigs, 73,655 transcripts with a median size (N50) of 1,818 bp and 33,238 unigenes with an N50 of 1,628 bp were assembled (Table 1, Dataset S1). Of the 33,238 unigenes, ∼54% were longer than 500 bp. Approximately 4,000 unigenes (12.16%) were longer than 2 kb. The total length of the assembled transcriptome is ∼32.4 Mbp (Table 1).

**Table 1:** Summary of *de novo* assembly of the *I. indigotica* transcriptome.

Feature	Number of features					Total length (bp)	N50 (bp)	Mean length (bp)
	<0.5 kb	0.5–1 kb	1–2 kb	>2 kb	Total			
Contig	1,167,979 (98.23%)	9,007 (0.76%)	8,132 (0.68%)	3,920 (0.33%)	1,189,038	96,133,270	94	81
Transcript	21,166 (28.74%)	16,575 (22.50%)	22,587 (30.67%)	13,327 (18.09%)	73,655	92,006,472	1,818	1,249
Unigene	15,195 (45.72%)	6,670 (20.07%)	7,331 (22.06%)	4,042 (12.16%)	33,238	32,381,334	1,628	974

In order to verify the quality of the assembly, a cDNAs fragment of seven randomly selected unigenes was amplified using unigene-specific primers and sequenced (Table S1 and S2). According to this experiment, firstly, an expected size of cDNA fragment was amplified for all seven unigenes (Figure S1); secondly, for each unigene, sequence of the amplified cDNA perfectly matched with that of assembled. These results suggest that the assembly is in high quality. At the same time, each primer set was used to amplify the corresponding genomic DNA (gDNA) of the seven unigenes (Dataset S2). For each unigene, the cDNA sequence matched well with its corresponding gDNA except some mismatches at one or both ends, which was most likely caused by not perfect reading at the beginning of the sequences during direct sequencing using one of the PCR primers (Figure S2). The gDNA sizes of two unigenes (Isatis indigotica 1223 and Isatis indigotica 5014) were the same as their corresponding cDNAs (Figure S1), suggesting that Isatis indigotica 1223 and Isatis indigotica 5014 contains no intron whereas the other five unigenes contain intron(s) in the amplified fragments (Figure S2).

### Annotation and classification of *I. indigotica* unigenes

To know the potential functions of the assembled unigenes, all 33,238 unigenes were subjected to blast search against various databases. First, these unigenes were searched against the NCBI non-redundant protein database (nr) using BLASTX with a cut-off E-value of 10^−5^. Since a relatively longer *k*-mer (*k* = 25) and strict criteria were used in the *de novo* assembly, the chance of mis-assembly was minimized. As a result, a high percentage of unigenes had a match in this database. Out of the 33,238 unigenes, 24,790 unigenes (74.6%) had a match, and 8,448 (25.4%) did not have a match. A portion of these un-matched unigenes might be unique to *I. indigotica*. Of the 24,790 unigenes with an orthologous match, 15,498 (62.5%) had a match with an annotated function, the remaining had a match only classified as hypothetical protein, predicted protein or putative protein. In addition, it is noteworthy that 27 unigenes matched with previously reported *I. tinctoria* genes.

Further blast search against other databases showed that 18,679, 24,794, 22,365, 7,707, 5,365 and 26,322 unigenes had a match in the SwissProt, TrEMBL, GO, COG, KEGG and nt (non-redundant nucleotide database) databases, respectively. In total, 28,184 unigenes had a match in at least one of the aforementioned databases, and 3,228 of them had a hit only in the nt database without detailed annotation (Table S3).

**Figure 1:**
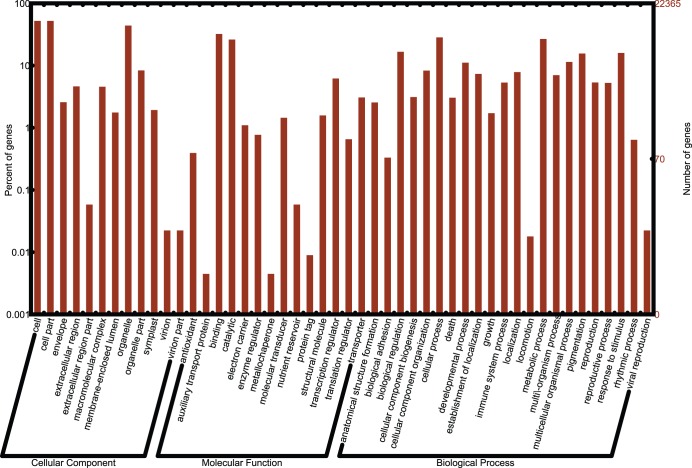
Histogram of GO classifications of the assembled *I. indigotica* unigenes. Results are summarized in three main GO categories: biological process, cellular component and molecular function.

To further evaluate the functions of the *I. indigotica* unigenes, 22,365 unigenes with a match in the GO database were classified based on their GO terms. It showed that these unigenes could be categorized into 48 functional sub-groups of the three main GO groups, *i.e.* molecular function, cellular component and biological process (Figure 1). The most frequent GO term in the groups of cellular component biological process, and molecular function were cell part (11,658 unigenes), binding (7,196 unigenes) and cellular process (6,330 unigenes), respectively.

**Figure 2:**
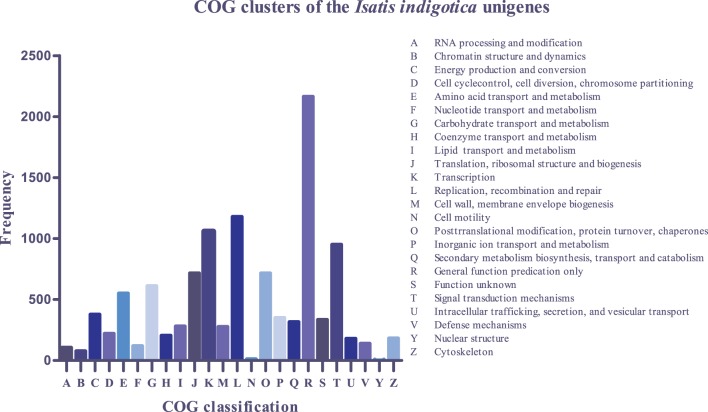
Histogram of COG clusters of the *I.indigotica* unigenes. Out of the 33,238 *de novo* assembled unigenes, 7,707 had a match in COG and were grouped into 24 clusters.

In addition, 7,707 of the total 33,238 *I. indigotica* unigenes could be classified into 24 clusters based on Clusters of Orthologous Groups (COG) analysis. Most of these classified unigenes belong to the cluster of “general function prediction” (2,163; 28.1%), which was followed by clusters of “replication, recombination and repair” (1179; 15.3%), “transcription” (1,058; 13.7%) and “signal transduction mechanisms” (950; 12.3%). The clusters represented by the least number of unigenes were “cell motility” (11; 0.14%), “nuclear structure” (2; 0.03%) and none was related to “extracellular structures” (0) (Figure 2).

### KEGG pathway mapping

**Figure 3:**
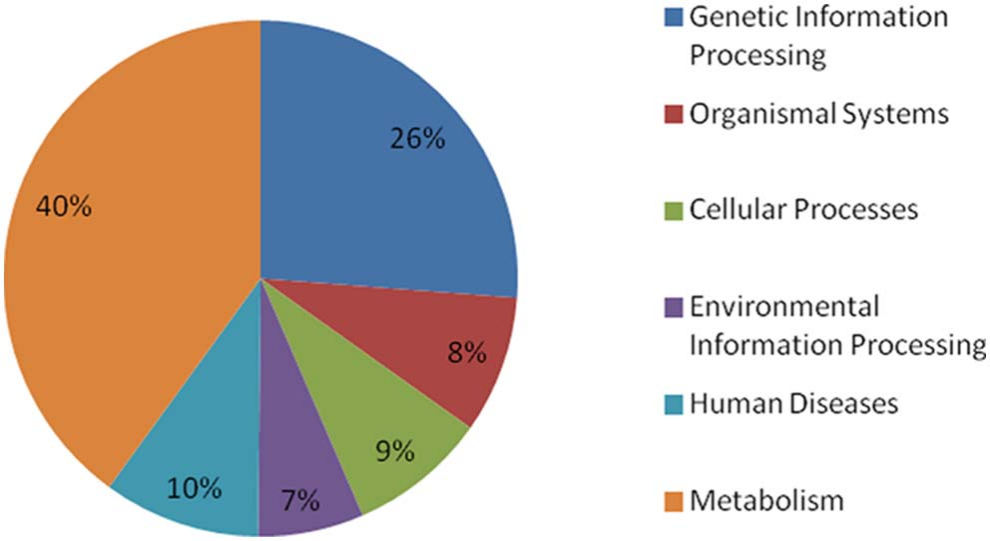
Pathway assignment based on KEGG mapping.

To identify the biological pathways represented by the unigenes assembled in this study, we compared all *I. indigotica* unigenes with that included in the KEGG database. In total, 5,398 unigenes could be assigned to 299 pathways that belong to six categories, including metabolism (40%), genetic information processing (26%), organismal system (8%), cellular processes (9%), environmental information processing (7%) and human diseases (10%) (Figure 3). The category with the largest number of unigenes was metabolism, which includes amino acid metabolism (122), biosynthesis of metabolites (837), degradation of metabolites (434), nucleotide metabolism (179), lipid metabolism (220), nitrogen metabolism (45) and biosynthesis of indole alkaloids (8). The unigenes with a potential role in metabolism of indole and its derivatives were listed in Table S3. The second largest category was genetic information processing, which contained 857 unigenes. High yield and content of the effective medicinal components, such as indole alkaloid and its derivatives, are the two major targets of *I. Indigotica* production. To achieve these goals, previous studies in *I. indigotica* have been focused on metabolism of nitrogen and secondary substances, such as indole alkaloids. Identification of 45 unigenes involved in nitrogen metabolism and 8 unigenes related to biosynthesis of indole alkaloids in this study provide foundation for molecular characterization of their roles in biosynthesis of indole alkaloids in *I. Indigotica* (Figure S3).

### Identification of SSRs in *I. indigotica* unigenes

**Figure 4:**
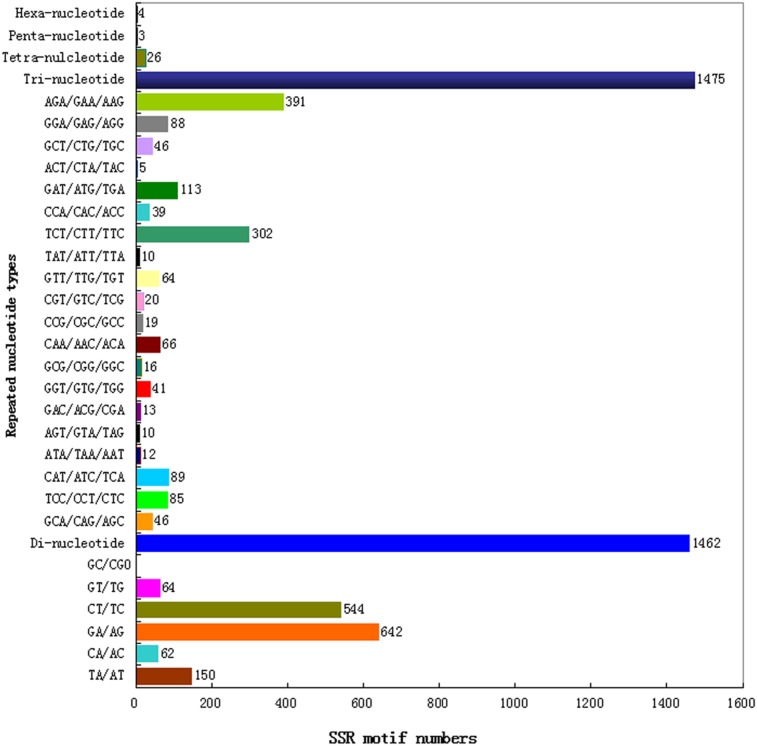
Distribution of different types of simple sequence repeats (SSRs) identified in the *I. indigotica* unigenes that are longer than 1000 bp.

Transcriptome is an important sequence resource for identification and development of molecular markers, such as SSR and single nucleotide polymorphism. Because only one genotype of *I. indigotica* was sequenced in this study, only SSR identification was performed. Unigenes with a length longer than 1 kb were searched for SSR markers using the MISA software. Of the 11,373 unigenes that are longer than 1 kb, 4,509 unigenes were found to contain 6,400 SSR markers (Table S4). Among these 4,509 unigenes, 1,378 contained two or more SSRs. The most frequent type of SSR was mono-nucleotide (3,430; 53.59%) but penta- and hexa-nucleotide types of SSRs were also identified (Table S4). Of the SSRs identified, 552 presented in a compound formation. According to the distribution of SSR motifs, (GA/AG)n, (CT/TC)n and (TA/AT)n were the three predominant types among the di-nucleotide SSRs, with a frequency of 43.91%, 37.21% and 10.26%, respectively. In the 20 types of tri-nucleotide SSRs, GAA (10.85%) was the most common SSR, followed by AGA (8.81%), TCT (8.41%) and AAG (6.85%) (Figure 4). So far there is no publically available SSR in *I. indigotica*, SSRs identified in this study are thus highly variable for studies of quantitative genetics and mapping of quantitative trait in *I. indigotica*.

## Discussion

Functional annotation and classification of transcriptome can provide clues on intracellular metabolic pathways and biological behaviors of genes. Insights on the functions of the *I. indigotica* unigenes were achieved by blast search against various databases. Among these databases, GO and COG are the most commonly used for functional classification of unigenes. COG is a database in which orthologous genes are classified and clustered. Every protein in COG is assumed to be evolved from an ancestor protein, and the whole database is built based on proteins of completely annotated genomes as well as their systematic evolutionary relationships with the orthologous proteins in bacteria and algae. GO is an international standardized gene functional classification system which offers a controlled vocabulary and strictly defined concept to comprehensively describe properties of genes and their products in any organism. With the help of GO functional classification, we could understand the distribution of gene function at the macro level and predict the potential physiological and molecular role of each unigene. COG and GO classifications revealed that the assembled *I. indigotica* unigenes have diverse molecular functions and are involved in a wide range of metabolic pathways (Figures 1, 2). In addition, by search against the KEGG database, we were able to assign a large number of unigenes into different metabolism pathways, including carbon metabolism, nitrogen metabolism, glucose metabolism and indole alkaloid metabolism (Figure 3). These results will facilitate molecular characterization of the genes involved in the pathways of interest.

Because of the economical and medicinal importance of *I. indigotica*, studies of *I. indigotica* have been emphasized on not only the pharmacological activity of chemical components but also the molecular function of the genes involved in biogenesis of the chemical components. However, only a couple of *I. indigotica* genes have been characterized in detail [33], [34]. Studies on the *LEA* (*LATER EMBRYOGENESIS ABUNDANT*) gene (*i.e*. *IiLEA*) of *I. indigotica* suggest that expression of *IiLEA* could be induced by environmental stresses, such as drought and salt treatments [33]. In this study, 8 unigenes were found to be associated with biosynthesis of indole and its derivatives. Studies on these genes could provide useful information for on secondary metabolism in *I. indigotica*. In addtion, 1,068 and 311 unigenes seem to be related to responses to salt stress and oxidation stress, respectively. Investigations on these unigenes may provide clues for the molecular mechanism of stress responses in cultivation of *I. indigotica*.

In summary, 33,238 unigenes were assembled in this study using the short reads obtained by sequencing of the leaf and root transcriptome of *I. indigotica*. Of these unigenes, 22,365 were associated with a GO annotation. The biological pathways involving some of these unigenes were also identified. To our knowledge, this is the first study on the transcriptome of *I. indigotica*. The unigenes presented in this study provide a substantial addition to the existing sequence resources of *I. indigotica* and are likely to promote studies on nitrogen metabolism, molecular mechanism of stress responses and secondary metabolism, such as indole alkaloids, quinoline and quinazolone, in *I. indigotica*.

## Materials and Methods

### Plant materials and RNA isolation

Seeds of *Isatis indigotica* Fort. cultivar SHX used in this study was sown at the Experimental Station of Nanjing Agricultural University (Nanjing, Jiangsu) on May 10^th^, 2012. Leaf and root samples were collected from two vegetative growth stages, namely the stages with 8∼10 leaves and 14∼16 leaves. These two stages were selected because the medicinal materials of Da-Qing-Ye (leaves) and Ban-Lan-Gen (roots) are collected at the 8∼16-leaf stage and 14∼16-leaf stage, respectively. Collected leaves and roots of *I. indigotica* were mixed and immediately frozen in liquid nitrogen and stored at −80°C until use. Total RNAs were isolated from the mixed sample using the Trizol plus kit (Biouniquer) and treated with DNase I to remove contaminated DNAs. The quality and integrity of the DNase I-treated RNA were analyzed using a 2100 Bioanalyzer (Agilent Technologies). Beads with oligo(dT) were used to isolate poly(A) mRNA from total RNA (Qiagen GmbH, Hilden, Germany).

### RNA-seq library construction and sequencing

RNA-seq library was constructed from mRNA using the Paired-End Sample Preparation Kit according to the manufacturer’s instructions and sequenced using the Illumina HiSeq 2000 (Illumina Inc., San Diego, CA, USA). The library was prepared from 200–250 bp (average size ∼230 bp) size selected cDNA fragments and was sequenced to generate 101-bp paired-end reads.

### Transcriptome *de novo* assembly and annotation of unigenes

NGSQC Toolkit (v2.3) [30] was firstly used to remove low quality reads, *i.e*. reads with 10% or more low quality bases (PHRED score <20). Transcriptome *de novo* assembly was carried out using the short read assembling software Trinity (http://trinityrnaseq.sourceforge.net/) [31]. Clean reads were assembled using the command of Trinity.pl with the following settings: –seqType fq –JM 100 G –left reads_1.fq –right reads_2.fq –CPU 30. Scaffolds produced by the Iinchworm module were termed as contigs, and sequences stored in file “Trinity.fasta” are treated as transcript. The standalone Blat software (The BLAST-like Alignment Tool, http://hgdownload.soe.ucsc.edu/admin/exe/linux.x86_64/blat) was used to cluster transcripts into clusters according to sequence similarities with parameters: -tilesize = 8 -stepsize = 5 (Dataset S3). The longest transcript in a clustering unit was selected as unigene. A unigene database was then constructed.

### Annotation and classification of unigenes

Unigenes were annotated by search (using BLASTX) against various protein databases, including nr (NCBI non-redundant protein database), Swiss-Prot, TrEMBL, COG and KEGG using a cut-off E-value of 10^−5^. Furthermore, unigenes were searched (using BLASTN) against the NCBI nucleotide database (nt) using a cut-off E-value of 10^−5^. Hits with the highest sequence similarity along with their protein functional annotations were retrieved. If the results from different databases conflicted to each other, a priority order of nr, Swiss-Prot, KEGG, COG and nt was followed for confidence. Assignment of unigenes to pathways was performed by search against the KEGG databases. The coding sequences of unigenes were determined based on their orthologous proteins. Unigenes that did not have a hit in any database were scanned using ESTScan [35] to find potential coding regions.

The Blast2 GO program was used to obtain GO annotations for the unigenes using a cut-off value of 10^−5^ [36]. This analysis mapped all of the annotated unigenes to GO terms in the database and counted the number of unigenes associated with each term. The WEGO software was then used to plot GO functional classification for the unigenes with a GO term hit to view the distribution of gene functions of the species at the macro level [37].

### Identification of SSR markers

The unigenes were scanned for microsatellites using the MISA (MIcroSAtellite identification tool) software (http://pgrc.ipk-gatersleben.de/misa/) with the default parameters. Perfect di-, tri-, tetra-, penta-, and hexa-nucleotide motifs were detected. The criteria for the SSRs are as following: mono-nucleotide type SSR requires a minimum of 10 repeats, di-nucleotide type SSR requires a minimum of 6 repeats, and the other type SSRs, including tri-, tetra-, penta-, and hexa-nucleotide, requires a minimum of 5 repeats.

### cDNA and gDNA amplification and sequencing for confirmation of unigenes assembled based on the RNA-seq data

Total RNA was isolated from leaves of the 8-leaf stage *I. indigotica* seedlings using the Trizol plus kit (Biouniquer). After treated with DNase I, 2 µg of total RNA was reverse-transcribed into cDNA by random primer using the Bu-SuperScript RT Kit (Biouniquer) according to the manufacturer’s instructions. cDNA fragments were then amplified in a Mastercycler (Eppendorf, Hamburg, Germany) using unigene-specific primers, which were designed using the Primer Premier 5 software. The primers used were listed in Table S1. The PCR reaction (20 µL) consists of: 2 µL of cDNA, 2 µL of 10×Buffer, 2 µL of forward and reverse primers (2 µM), 2 µL of MgCl_2_ (2.5 mM), 0.2 µL of Taq DNA Polymerase (5000 U/mL), 2 µL of dNTPs (2 mM) and 9.8 µL of ddH_2_O. Amplification conditions were: 95°C, 5 min; 35 cycles of 95°C, 30 s; 55°C, 30 s; 72°C, 30 s; and finally elongation at 72°C for 5 minutes. The PCR products were separated on 1% agarose gel, excised and gel purified and then sequenced directly at Invitrogen (Shanghai, China). The corresponding genomic DNA fragments of the cDNAs were amplified using DNA extracted with the CTAB method and purified and sequenced as aforementioned. cDNAs and their corresponding genomic DNAs were aligned to find intron(s) in the unigenes.

## Supporting Information

Figure S1 **Verification of the assembled unigenes by cDNA cloning and sequencing.** cDNA and genomic DNAs of seven randomly selected unigenes were amplified and sequenced. In all seven cases, the assembled unigene sequences were confirmed. This Figure shows the size of cDNAs and their corresponding genomic DNAs of the seven unigenes. For each pair (e. g. 1 and 1′), the first and second lane represent cDNA and genomic DNA, respectively. M: DNA ladder.
(DOC)

Figure S2 **Alignment of the amplified genomic DNA and cDNA of the seven selected unigenes.** The alignments were generated by DNAMAN. Matched nucleotides were highlighted in blue background.
(DOC)

Figure S3 **KEGG analysis of indole alkaloids biosynthesis.**
(DOC)

Table S1 **Information of the seven selected unigenes.**
(DOC)

Table S2 **List of unigenes with a potential role in biosynthesis of indole and its derivatives.**
(DOC)

Table S3 **Annotation and classification of 28184** ***I. indigotica*** **unigenes.**
(XLS)

Table S4 **Statistics of the SSRs identified in the** ***I. indigotica*** **unigenes.**
(DOC)

Dataset S1 **Assembled sequences of** ***I. indigotica*** **unigenes.**
(FASTA)

Dataset S2 **Amplified genomic DNA and cDNA sequences of the unigenes selected for verification.**
(FASTA)

Dataset S3 **Information of** ***Isatis indigotica*** **clusters and transcripts.**
(TXT)
