# Supplementary figures and images for: High-Throughput Sequencing and De Novo Assembly of the Isatis indigotica Transcriptome
Source: PLoS One. 2014 Sep 26;9(9):e102963. doi: 10.1371/journal.pone.0102963 (PMC4178013; doi:10.1371/journal.pone.0102963)

**Figure S1** Verification of the assembled unigenes by cDNA and gDNA cloning and sequencing


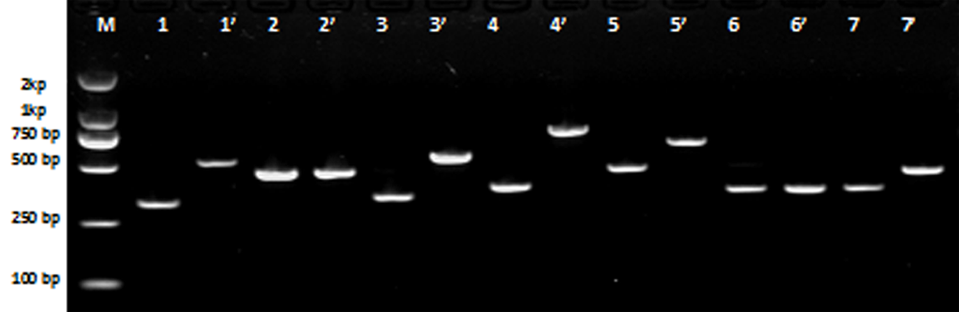

Supplement: Figure S1 — Verification of the assembled unigenes by cDNA cloning and sequencing. cDNA and genomic DNAs of seven randomly selected unigenes were amplified and sequenced. In all seven cases, the assembled unigene sequences were confirmed. This Figure shows the size of cDNAs and their corresponding genomic DNAs of the seven unigenes. For each pair (e. g. 1 and 1′), the first and second lane represent cDNA and genomic DNA, respectively. M: DNA ladder. (DOC) [file pone.0102963.s001.doc]

**Figure S3** KEGG analysis of indole alkaloids biosynthesis


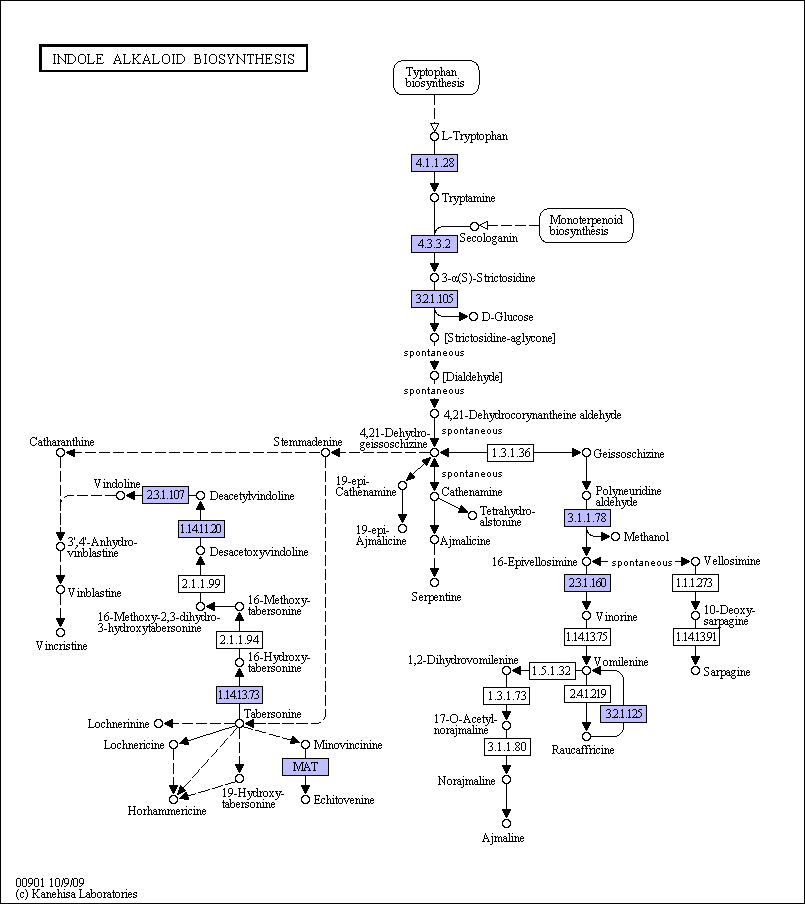

Supplement: Figure S3 — KEGG analysis of indole alkaloids biosynthesis. (DOC) [file pone.0102963.s003.doc]
