# Supplementary material for: High-Throughput Sequencing and De Novo Assembly of the Isatis indigotica Transcriptome
Source: PLoS One. 2014 Sep 26;9(9):e102963. doi: 10.1371/journal.pone.0102963 (PMC4178013; doi:10.1371/journal.pone.0102963)

**Figure S2** Alignment of genomic DNA and cDNA of the seven selected unigenes. The alignments were generated by DNAMAN.

1. Isatis_indigotica_1223


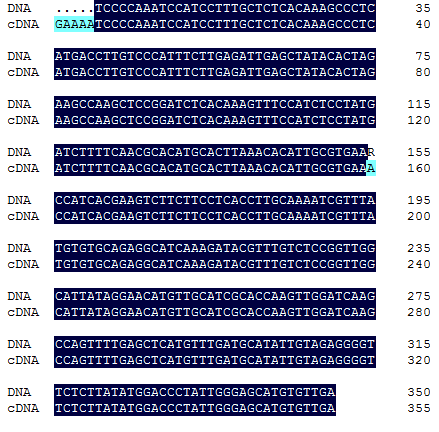


2. Isatis_indigotica_4199


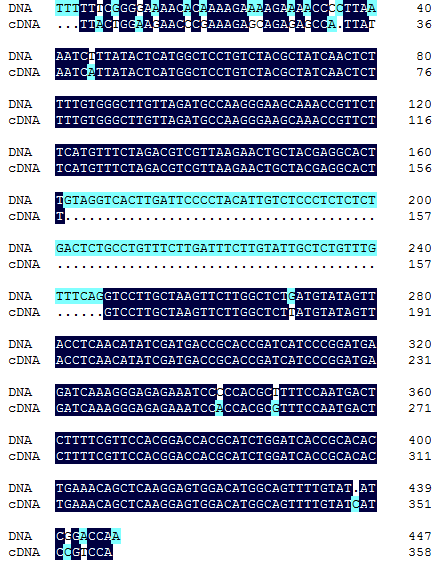


3. Isatis_indigotica_5014


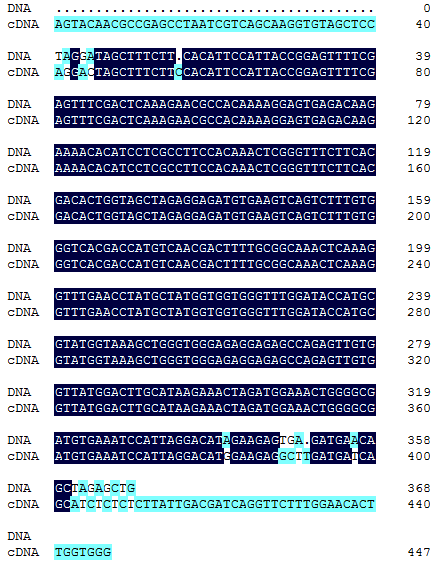


4. Isatis_indigotica_6218


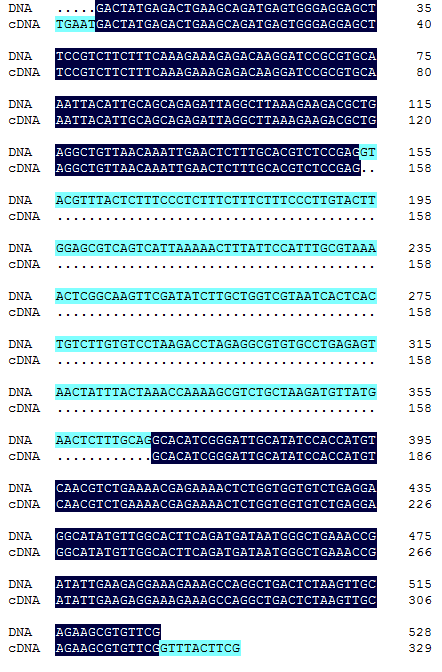


5. Isatis_indigotica_8821


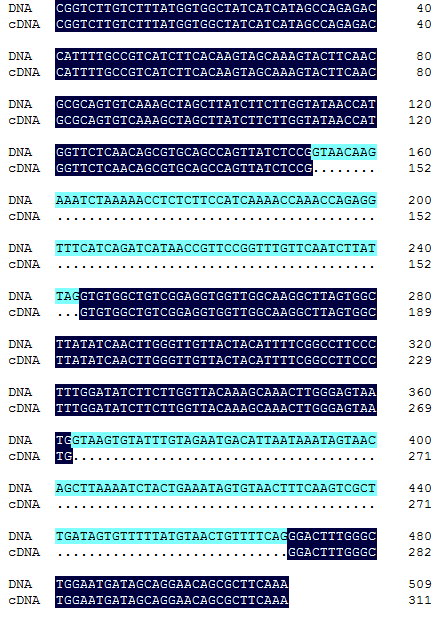


6. Isatis_indigotica_15680


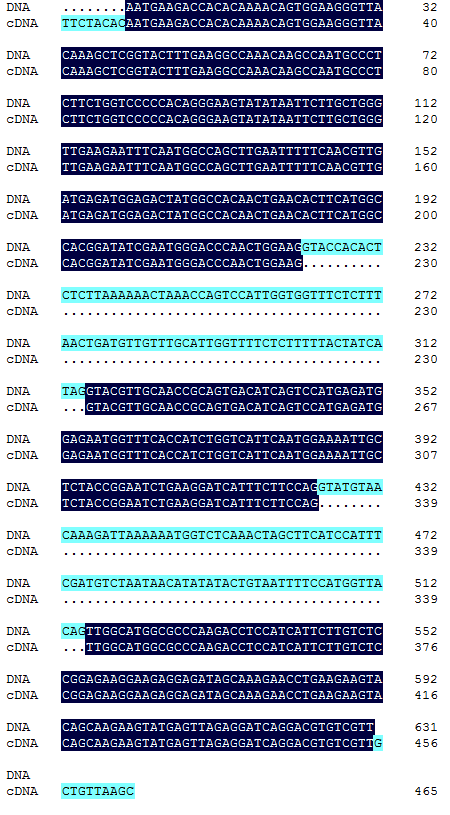


7. Isatis_indigotica_15731


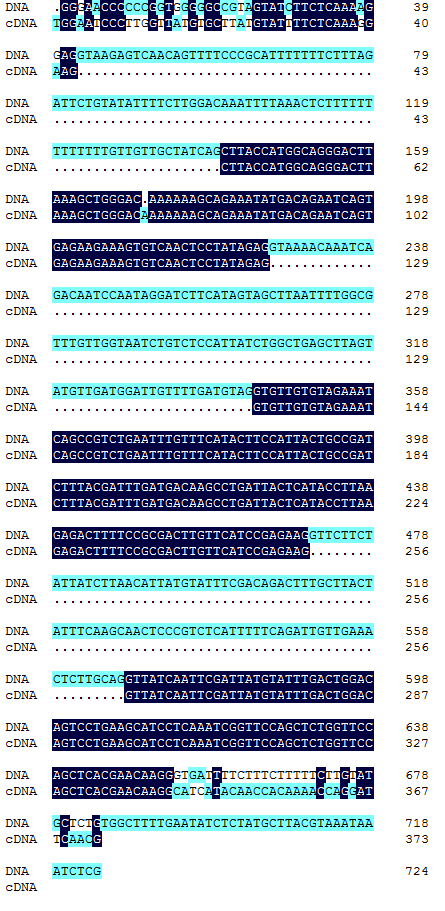

Supplement: Figure S2 — Alignment of the amplified genomic DNA and cDNA of the seven selected unigenes. The alignments were generated by DNAMAN. Matched nucleotides were highlighted in blue background. (DOC) [file pone.0102963.s002.doc]
