# Supplementary material for: High-Throughput Sequencing and De Novo Assembly of the Isatis indigotica Transcriptome
Source: PLoS One. 2014 Sep 26;9(9):e102963. doi: 10.1371/journal.pone.0102963 (PMC4178013; doi:10.1371/journal.pone.0102963)
Supplement: Table S1 — Information of the seven selected unigenes. (DOC) [file pone.0102963.s004.doc]

Table S1 Information of the seven selected unigenes

| No. | Unigene  ID | Nucleotide sequence hit in GenBank (similarity%) | | Primers | Length of the PCR product (bp) |
| --- | --- | --- | --- | --- | --- |
| 1 | Isatis_indigotica_1223 | | GAKE01013824.1 (91%),  *Thlaspi arvense* | Forward: 5' CGAAAATCCCCAAATCCA 3'  Reverse: 5' TCAACACATGCTCCCAAT 3' | 356 |
| 2 | Isatis_indigotica_4199 | | GAKE0101009754.1 (93%), *Thlaspi arvense* | Forward: 5' TTACTGGAAGAACCCGAAAG 3'  Reverse: 5' TGGACGGATGATACAAAACT 3' | 358 |
| 3 | Isatis_indigotica_5014 | | NM125709.4 (84%), *Arabidopsis thaliana* | Forward: 5' AGTACAACGCCGAGCCTAA 3'  Reverse: 5' CCCACCAAGTGTTCCAAAG 3' | 447 |
| 4 | Isatis_indigotica_6218 | | GAKE01018968.1 (85%),  *Thlaspi arvense* | Forward:5'TGAATGACTATGAGACTGAAGC 3'  Reverse: 5' CGAAGTAAACCGAACACG 3' | 329 |
| 5 | Isatis_indigotica_8821 | | GAKE01002094.1 (94%), *Thlaspi arvense* | Forward: 5' CGGTCTTGTCTTTATGGTGG 3'  Reverse: 5' TTTGAAGCGCTGTTCCTG 3' | 311 |
| 6 | Isatis_indigotica_15680 | | NM122646.3 (90%), *Arabidopsis thaliana* | Forward:5'TTCTACACAATGAAGACCACAC 3'  Reverse: 5' GCTTAACAGCAACGACACG 3' | 465 |
| 7 | Isatis_indigotica_15731 | | GAKE0101000380.1 (92%), *Thlaspi arvense* | Forward: 5' TGGAATCCCTTGGTTATGTGC 3'  Reverse: 5' CGTTGAATCCTGGTTTTGTG 3' | 373 |
