# Supplementary material for: High-Throughput Sequencing and De Novo Assembly of the Isatis indigotica Transcriptome
Source: PLoS One. 2014 Sep 26;9(9):e102963. doi: 10.1371/journal.pone.0102963 (PMC4178013; doi:10.1371/journal.pone.0102963)
Supplement: Table S2 — List of unigenes with a potential role in biosynthesis of indole and its derivatives. (DOC) [file pone.0102963.s005.doc]

Table S2 The unigenes with potential role in metabolism of indole and its derivates

| No. | Unigene  ID | Function prediction | Nucleic acid Length(bp) |
| --- | --- | --- | --- |
| 1 | Isatis_indigotica_4516 | indoleacetic acid biosynthetic process | 371 |
| 2 | Isatis_indigotica_6439 | L-serine hydro-lyase (adding indole, L-tryptophan-forming) activity | 1862 |
| 3 | Isatis_indigotica_8669 | indole-3-glycerol-phosphate synthase activity | 1416 |
| 4 | Isatis_indigotica_9081 | indoleacetic acid biosynthetic process | 1812 |
| 5 | Isatis_indigotica_10759 | indoleacetic acid biosynthetic process | 1296 |
| 6 | Isatis_indigotica_10862 | indole-3-glycerol-phosphate lyase activity | 1654 |
| 7 | Isatis_indigotica_29073 | indoleacetic acid biosynthetic process | 1570 |
| 8 | Isatis_indigotica_33223 | indoleacetic acid biosynthetic process | 1099 |
