# Supplementary material for: High-Throughput Sequencing and De Novo Assembly of the Isatis indigotica Transcriptome
Source: PLoS One. 2014 Sep 26;9(9):e102963. doi: 10.1371/journal.pone.0102963 (PMC4178013; doi:10.1371/journal.pone.0102963)
Supplement: Table S4 — Statistics of the SSRs identified in the I. indigotica unigenes. (DOC) [file pone.0102963.s007.doc]

Table S4 Statistics of the SSRs identified in the *I. indigotica* unigenes

| Category | Number | Percentage of the total number of identified SSRs (%) |
| --- | --- | --- |
| Total number of unigenes examined (>1 kb) | 11,373 |  |
| Total size of the examined sequences (bp) | 22,789,565 |  |
| Total number of the identified SSRs | 6,400 |  |
| Number of SSR-containing unigenes | 4,509 |  |
| Number of unigenes containing ≥ 2 SSRs | 1,378 |  |
| Number of SSRs present in compound formation | 552 |  |
| Mono-nucleotide | 3,430 | 53.59 |
| Di-nucleotide | 1,462 | 22.84 |
| Tri-nucleotide | 1,475 | 23.05 |
| Tetra-nucleotide | 26 | 0.41 |
| Penta-nucleotide | 3 | 0.05 |
| Hexa-nucleotide | 4 | 0.06 |
